# Higher energy consumption in the evening is associated with increased odds of obesity and metabolic syndrome: findings from the 2016-2018 Korea National Health and Nutrition Examination Survey (7th KNHANES)

**DOI:** 10.4178/epih.e2023087

**Published:** 2023-09-19

**Authors:** Sarang Jeong, Hajoung Lee, Sukyoung Jung, Jee Young Kim, Sohyun Park

**Affiliations:** 1 The Korean Institute of Nutrition, Hallym University, Chuncheon, Korea; 2 EyeLight Data Science Laboratory, Seoul National University College of Medicine, Seoul, Korea; 3 Department of Ophthalmology, Seoul National University Hospital, Seoul, Korea; 4 Chungnam National University Hospital Biomedical Research Institute, Daejeon, Korea; 5 Department of Epidemiology, Milken Institute School of Public Health, The George Washington University, Washington, DC, USA; 6 National Food Safety Information Service, Seoul, Korea; 7 Department of Food Science and Nutrition, Hallym University, Chuncheon, Korea

**Keywords:** Meal times, Circadian rhythm, Eating behavior, Metabolic syndrome, Obesity

## Abstract

**OBJECTIVES:**

Chrono-nutrition emphasizes meal timing in preventing obesity and metabolic disorders. This study explores the impact of temporal dietary patterns (TDPs) on obesity and metabolic syndrome (MetS) in Korean adults aged 20 years to 65 years.

**METHODS:**

We utilized dynamic time warping method and Kernel k-means clustering to investigate diet quality and the odds ratios (ORs) of obesity and MetS with different TDPs using data from the 7th Korea National Health and Nutrition Examination Survey.

**RESULTS:**

Participants were divided into three groups based on relative energy intake over 24 hours. After adjusting for age and gender, Cluster 3 (with the highest proportion of energy intake in the evening) had the lowest Healthy Eating Index scores compared to other clusters. Following adjustment for key covariates, Cluster 3 showed the highest values for body mass index, waist circumference, blood pressure, total cholesterol, and triglycerides. Compared to Cluster 1 (with a lower proportion of energy intake in the evening), Cluster 2 and Cluster 3 had ORs for obesity of 1.12 (95% confidence interval [CI], 0.97 to 1.30) and 1.19 (95% CI, 1.03 to 1.37), respectively. For MetS, the ORs were 1.26 (95% CI, 1.08 to 1.48) and 1.37 (95% CI, 1.17 to 1.61) when comparing Cluster 2 and Cluster 3 to Cluster 1.

**CONCLUSIONS:**

This study reveals that individuals with higher energy intake in the evening have increased odds of obesity and MetS, even after adjusting for major covariates, including age and total energy intake.

## GRAPHICAL ABSTRACT

[Fig f2-epih-45-e2023087]

## INTRODUCTION

Chrono-nutrition, which refers to the study of the temporal aspect of nutrition, has gained considerable attention in recent years [1,2]. This field recognizes the significance of aligning food intake with the body’s circadian rhythms, which regulates various physiological functions such as the sleep-wake cycle [3], hormone secretion [4], and metabolism [5]. Disruption in the 24-hour circadian rhythm due to factors such as shift work, irregular sleep patterns, or irregular eating habits are linked to several health issues, including obesity, diabetes, and cardiovascular disease [6]. Irregular eating habits, such as skipping meals or consuming meals late at night, may disrupt the circadian rhythm and lead to metabolic disorders [7]. Conversely, consuming a well-balanced breakfast in the morning is associated with improved blood sugar control and weight management [8]. Furthermore, chrono-nutrition emphasizes the importance of meal frequency along with meal timing [9].

Research has indicated that consuming energy in the early time of the day may be beneficial in preventing insulin resistance and obesity compared to consuming energy in the late time [10]. Moreover, a study has shown that individuals with regular, energy-balanced mealtimes have a lower body mass index (BMI) and waist circumference (WC) compared to those with irregular eating occasions and a non-balanced intake of energy [11]. As a result, investigating the timing of the highest proportion energy intake during the day and meal frequency could be of significant importance for preventing or managing obesity and metabolic syndrome (MetS). Moreover, studying dietary habits in relation to human circadian rhythms could be crucial for understanding weight reduction or management strategies among individuals prone to weight gain.

Studies conducted with American adults have revealed that individuals practicing Time-Restricted Eating, characterized by equal intervals of balanced energy intake within limited hours of the day, had significantly lower average BMI, WC, and obesity probability when compared to those with irregular energy intake throughout the day [12,13]. For Korean adults, research findings suggest that overall dietary patterns, including energy distribution throughout the day, meal frequency, and sleep duration, are associated with cardiovascular and metabolic risks [14,15]. To develop effective interventions and policies that promote healthy eating and prevent chronic diseases, it is vital to understand the relationship between time-related dietary patterns and health outcomes among diverse populations [16].

In addition, various statistical methods can be applied to capture time-related dietary habits as risk factors for body adiposity and metabolic disorders. However, there is still a scarcity of evidence using various statistical methods to understand the association between temporal dietary patterns (TDPs) and health outcomes [15]. Therefore, this study aimed to investigate the general characteristics, Healthy Eating Index (HEI), the odds of obesity and MetS among groups with different TDPs features, using a dynamic time warping (DTW) method and Kernel k-means clustering. This study population comprised Korean adults aged 20 years to 65 years, based on the data from the 2016-2018 Korea National Health and Nutrition Examination Survey (KNHANES).

## MATERIALS AND METHODS

### Analytical data

The KNHANES [17] is a comprehensive public health survey that provides representative data for Korea on various health and socioeconomic indicators. This cross-sectional survey is conducted annually and serves as a basis for developing health policies and calculating nationally representative health and nutritional statistics for the Korean population. The 7th KNHANES provides the most recent data that represent the Korean population with food consumption data that have timing of each eating occasion. For the 7th KNHANES conducted from 2016-2018, the survey was based on city/province, *dong/eup/myeon*, and housing type (single-family homes, apartments). In the first year (2016) of the 7th survey, 192 survey districts were selected using a systematic sampling method, and 23 sample households were selected among appropriate households, including foreign households within the sample survey districts. All household members over the age of 1 year who met the appropriate household member requirements were selected as survey subjects within the sample households.

### Study population and characteristics

This study analyzed data from KNHANES collected between 2016-2018, which included a total of 8,147 Korean adults aged between 20 years and 65 years old. The data included information on gender, age, socioeconomic status, health status, 24-hour dietary recall, anthropometric measurements, and laboratory data. Participants who had unrealistic daily energy intake (less than 500 kcal or over 5,000 kcal) or serious health problems that could affect their food intake, as well as pregnant and breastfeeding women, were excluded from the study. Education levels were classified into three categories: less than middle school, high school, and over college. Household incomes were divided into quartile groups: lowest, lower-middle, upper-middle, and highest income groups. Furthermore, individuals who reported daily or occasional smoking were considered current smokers, and participants engaging in aerobic exercise were included if they reported engaging in moderate-intensity physical activity for more than 2 hours and 30 minutes, high-intensity physical activity for more than 1 hour and 15 minutes, or a combination of moderate and high-intensity physical activities for an appropriate duration per week for each activity. In cases where participants indicated a high level of perceived stress and reported consuming one or more alcoholic drinks per month in the past year, both responses were included.

### Anthropometric parameters and laboratory measurements

This study analyzed data on anthropometric and laboratory parameters. Anthropometric measurements included height, weight, BMI, hip circumference and WC, and systolic blood pressure (SBP) and diastolic of blood pressure (DBP). Anthropometric measurements and blood pressure (BP) assessments were conducted by skilled experts from the Korea Centers for Disease Control and Prevention. For weight measurements, a correction of -0.5 kg was applied when participants were not wearing examination attire. BP measurements were taken based on the average arm length corresponding to the height of the heart (83 cm for men and 81 cm for women) as a reference, and more detailed measurement procedures can be found in the 7th KNHANES [17]. Laboratory measurements included fasting blood glucose (FBG), triglycerides (TGs), total cholesterol (T-chol), high-density lipoprotein cholesterol (HDL-chol), and low-density lipoprotein cholesterol (LDL-chol). Taking these factors into account, MetS was diagnosed according to the adult criteria of the International Diabetes Federation [18], which are described in the Supplementary Material 1.

### Dietary assessment

To define TDPs using meal timing and energy intake proportion, this study utilized the 24-hour dietary recall data from KNHANES. The 24-hour dietary recall encompassed information on the types, quantities, and timing of all food consumed during breakfast, lunch, dinner, and snacks, along with the corresponding calorie and nutrient intake. The data was then processed by converting the volume of food into weight and dishes into food ingredients, and food into energy and nutrients. The reported dietary information was linked to Korea Rural Development Administration for National Standard Food Ingredients database [17]. In this study, the HEI was used to assess the quality of individuals’ diets [19]. Briefly, the HEI was calculated based on data collected through the 24-hour dietary recall. The HEI assigned scores based on the consumption of various food groups. It classified the components into adequacy items, moderation items, and balance of energy items. Adequacy items included breakfast, whole grains, fruits (any type and fresh only), vegetables (any type excluding pickled), protein sources, and dairy products. Moderation items included the sodium and the percentage of energy of saturated fat, and sugar. Balance of energy items included the energy ratio of carbohydrates and fats, as well as energy adequacy. Higher scores in HEI means better dietary quality.

### Temporal dietary patterns

The TDPs in this study was obtained by performing Kernel k-means clustering based on DTW distance using time series data representing the proportion of energy intake of 8,147 individuals over 24 hours. To perform Kernel k-means clustering using time series data, the distance between time series data must be calculated, and the common distance is the Euclidean distance, which measures the distance between two-time series at the same point in time [20]. However, the accuracy of similarity calculation using the Euclidean distance decreases significantly in situations where the time axis is not synchronized or these is a large difference in scale [21]. Additionally, it is impossible to calculate when the length of time series vector is different [22]. The purpose of this study is not to obtain clusters that consume the same amount of calories in a day, but to obtain clusters that have similar intake trends over time. In the analysis of energy intake trends, all participants’ food intake, whether meals or snacks, recorded over a 24-hour period, was taken into consideration. The analysis included all mealtimes that provided energy, and the energy consumed during the 24-hour period was evaluated as a relative proportion of the total calories consumed by individuals throughout the day.

Therefore, instead of using the Euclidean distance, the DTW distance, which minimizes the distance between two measured time series data by moving in the direction of minimizing the cumulative distance [23], is used. The data obtained in each cluster has similar energy intake trends, and their meaning can be interpreted through comparison of each cluster. The cluster initial value of the Kernel k-means clustering was set from 2 to 10, and the optimal number of clusters was determined using cluster validity indices as the performance measure. In this study, 6 representative indices, including Silhouette index [24], Dunn index [25], Calinski-Harabasz index [26], Score function [27], Davies-Bouldin index [28], and Modified Davies-Bouldin index [29], were used as cluster validity indices (Supplementary Material 2). Detailed information about the method used to determine the optimal number of clusters is provided in the Supplementary Material 3.

### Statistical analysis

Statistical analysis was performed using Stata version 17.0 (Stata Corp., College Station, TX, USA). For the comparison of categorical variables (e.g., gender, education, and current smokers), a chi-squared test was conducted. Data are presented as mean± standard deviation values and numbers (percentage). All statistical analyses accounted for the complex survey design. Generalized linear model (GLM) was used to examine mean differences of key variables between clusters and post-hoc analyses were performed with Bonferroni adjustment for comparison in mean values among clusters. Logistic regression was employed to evaluate the association between obesity and MetS in each TDPs cluster, after adjusting for covariates including age, gender, smoking, drinking, participation in aerobic exercise, total energy intake and HEI. A p-value less than 0.05 was considered statistically significant.

### Ethics statement

The study protocol was approved by the Institutional Review Board (IRB) of Hallym University (IRB No. HIRB-2021-087-R-CR). Informed consent for the use of secondary data was waived by the IRB.

## RESULTS

### Characteristics of three clusters of temporal dietary patterns defined by a dynamic time warping method

**Figure 1.**
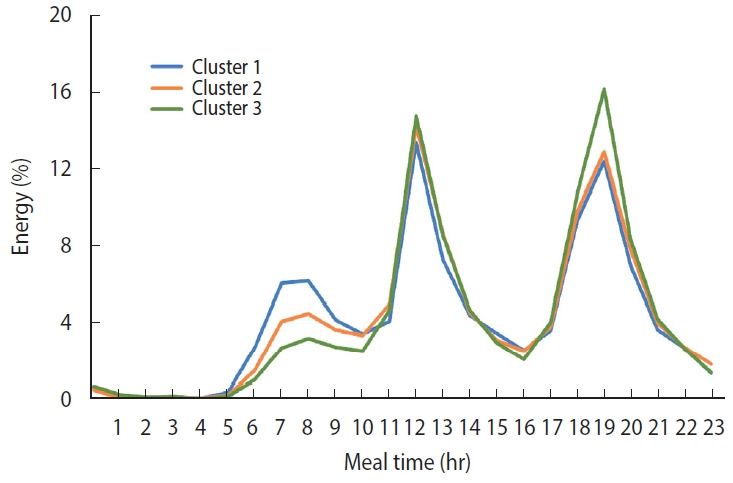
The daily energy intake distribution over 24 hours in three clusters defined by a dynamic time warping method. Cluster 1 had a higher percentage of energy intake in the morning, Cluster 2 had a higher percentage of energy intake in the lunch, and Cluster 3 had a higher percentage of energy intake in the evening.

The comparison of the peak event of daily energy intake over 24 hours in each cluster is shown in Figure 1. This curve shows the time-based energy intake patterns of the three clusters. Participants in Cluster 1 had a higher percentage of energy intake in the morning (6-9 a.m.) than the other clusters, while Cluster 3 had a higher percentage of energy intake in the evening (6-8 p.m.) compared to Clusters 1 and 2.

Table 1 illustrates the characteristics of three clusters of TDPs defined by a DTW method. The number of participants in each cluster was 3,334, 2,581, and 2,232, respectively. Cluster 1 tended to consume a larger breakfast and smaller dinners compared to the other clusters. On the other hand, participants in Cluster 3 had little breakfast and consumed a large amount of energy in the evening. Cluster 2 had some breakfast and smaller dinners than Cluster 3.

### Characteristics of participants according to temporal dietary patterns

The characteristics of participants in each cluster are shown in Table 2. The gender ratio in each cluster was 51.6, 54.0, and 55.6 for men and 48.4, 46.0, and 44.4 for women (p= 0.035). The age groups were divided into 20-34 years, 35-49 years, and 50-65 years, and the age group with the highest percentage was 50-65 years old in Cluster 1 and 35-49 years old in Clusters 2 and 3 (p< 0.001). Cluster 3 had the highest percentage of participants with higher education levels (p< 0.001). The percentage of current smokers, drinkers, and people who felt stressed were the highest in Cluster 3 (p< 0.001, < 0.001, 0.014, respectively). The household income level and people who participated in aerobic exercise did not show statistically significant differences among the three clusters.

### Nutritional intake and Healthy Eating Index according to temporal dietary patterns

Table 3 presents the nutritional intake in three clusters of TDPs. Total energy was statistically significantly different among three clusters (p<0.001). Carbohydrates (p<0.001), total sugars (p<0.001), fat (p< 0.001), saturated fat (p< 0.001), sodium (p= 0.033), sodium density per 1,000 kcal (p= 0.012), fiber (p< 0.001), and fiber density per 1,000 kcal (p< 0.001) were also statistically significantly different among three clusters. However, the protein was not significantly different among the three clusters.

The HEI scores were also statistically different in three clusters of TDPs. The total HEI and the subscores of adequacy, moderation, and balance of energy were significantly lower in Cluster 3 than in the other two clusters (p < 0.001, respectively). Similar results were observed for the breakfast (p< 0.001), whole grain (p< 0.001), any type of fruits (p< 0.001), fresh fruits (p< 0.001), any type vegetables (p < 0.001), vegetables excluding pickled (p= 0.001), protein sources (p= 0.019), dairy products (p< 0.001) of adequacy items, saturated fat (p< 0.001) and sodium (p= 0.031) of moderation items, and carbohydrates, fat, and energy adequacy (p< 0.001, respectively) of a balance of energy items. These subscores were also statistically significantly different among the clusters, with Cluster 1 having the highest scores.

### The metabolic indicators according to three clusters in temporal dietary patterns

The metabolic indicators of three clusters are shown in Table 4. The BMI, WC, SBP, DBP, TGs, and T-chol were statistically significantly different among the three clusters (p= 0.001, 0.001, 0.001, < 0.001, 0.001, and 0.005, respectively). The mean values of these indicators except DBP were the highest in Cluster 3. The FBG, LDL-chol, and HDL-chol were not significantly different in three clusters.

### The odds ratios of obesity and metabolic syndrome of three clusters of temporal dietary patterns

The ORs for obesity and MetS across the three clusters are presented in Table 5. Logistic regression analysis revealed that compared to Cluster 1, Clusters 2 and 3 had odds for obesity were 1.12 and 1.19 times higher, respectively, with 95% confidence intervals (CIs) of 0.97 to 1.30 and 1.03 to 1.37, respectively. Similarly, the odds for MetS of Clusters 2 and 3 were 1.26 and 1.37 times higher than Cluster 1, with 95% CI of 1.08 to 1.48 and 1.17 to 1.61, respectively. Cluster 2 showed higher odds of central obesity, elevated TGs, low HDL-chol, elevated blood glucose, and elevated BP compared to Cluster 1. For Cluster 3, the odds were higher in all these variables compared to Cluster 1.

## DISCUSSION

Employing Kernel k-means clustering with DTW, this study examined time-related eating patterns in 8,147 Korean adults. Cluster 3, characterized by a higher proportion of energy intake in the evening, showed lower HEI scores, elevated obesity odds, and less favorable metabolic markers compared to Cluster 1, which had a higher proportion of energy intake in the morning. These relationships remained significant after adjusting for key covariates, consistent with earlier research linking food intake timing to health outcomes.

Prior research highlighted links between health outcomes and dietary intake, assessed through methods like 24-hour dietary recalls [30] and food frequency questionnaire [31]. However, these methods have overlooked eating timing [32]. Chrono-nutrition, a growing field, has emphasized the impact of food timing on metabolic indicators [33]. Earlier studies suggested that consuming food earlier in the day optimizes nutrient absorption, metabolism [9,34], and health while aiding weight loss [35] and reducing MetS risk [36]. This study reinforces these findings.

The DTW method analyzes similarity between time sequences of varied lengths [37]. Applied here, it studies TDPs using Kernel k-means to linearly arrange time and energy intake data, useful for large populations [38]. This study’s novelty lies in being the first to comprehensively investigate TDPs’ link to obesity and MetS using Korean data, addressing limitations of previous studies [39,40].

The study’s primary findings highlight that Cluster 3, marked by a higher proportion of energy intake later in the day, exhibited higher odds of obesity and MetS. This relationship was statistically significant even after adjusting for key variables, usually regarded as main risk factors for body adiposity and metabolic disorders, such as age, education level, exercise, total energy intake, and diet quality. As the proportion of energy intake increased later in the day, participants showed higher ORs of obesity and MetS. As observed in studies by Bo et al. [41] evening meals yield lower resting metabolic rates compared to breakfast, resulting in increased blood sugar and insulin responses. This may explain one of the reasons for the higher odds of obesity and MetS among individuals with late meals shown in this study.

Previous research has also suggested the close relationship between meal timing, circadian rhythm, metabolism, and nutrition. One study found that eating late slowed down the period circadian regulator 2 (PER2) rhythm, a protein that plays a critical role in regulating the circadian rhythm, present in adipose tissue, and these changes may increase the risk of obesity [42]. The circadian rhythm plays a role in regulating physiological functions and maintaining the homeostasis of the human metabolic system [43]. Furthermore, previous studies have suggested that sleep patterns, meal timing, and meal composition may mediate these effects [44]. Moreover, a study has shown that delayed meals can also lead to a delay in the PER2 rhythm [45].

It is concerning that Cluster 3 had a higher proportion of younger participants. Data from the National Health Insurance Corporation in Korea indicates a faster rise in obesity rates, notably among young people in their 20s, increasing from 32.2% in 2009 to 39.6% in 2019 [44]. Until recently, dietary guidelines usually focus on the intakes of certain food groups and nutrients. As more evidence accumulate, dietary guidelines emphasizing the importance of eating timing may be needed to be integrated into nutrition education and public policies.

This research adds to chrono-nutrition by examining TDP-health associations in Korean diets. Cross-sectional design hinders causal inferences, and results might not extend to diverse ethnicities. Nevertheless, this innovative analysis of TDPs and their links to nutrition, obesity, and MetS among Koreans should be noteworthy and followed by future studies with more robust research designs such as prospective cohort and intervention studies. This will help to further elucidate the relationship between eating timing and health outcomes.

In conclusion, individuals with higher proportion of energy intake in the evening are associated with unhealthy behaviors, lower diet quality, and an elevated odds of obesity and MetS. These findings can be used in understanding the significance of TDP in shaping health outcomes.

## Figures and Tables

**Figure f2-epih-45-e2023087:**
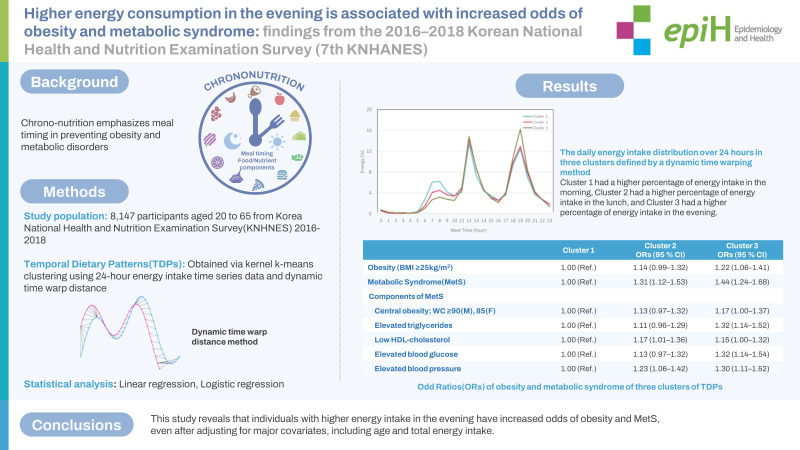


**Table 1.** Characteristics of three clusters of temporal dietary patterns defined by a dynamic time warping method (n=8,147)^1^

Characteristics	Cluster 1 (n=3,334, 38.8%)	Cluster 2 (n=2,581, 32.6%)	Cluster 3 (n=2,232, 28.6%)
Temporal patterns	Higher caloric intake during morning time and lower caloric intake during evening time compared to other clusters	Smaller caloric intake during morning time and higher caloric intake during evening time compared to Cluster 1	Smallest caloric intake during morning time and largest caloric intake during evening time compared to other clusters
Time-specific energy intake distribution (%)			
Morning time (0:00-10:59)	24.4	18.6	14.2
Day time (11:00-16:59)	35.4	38.3	38.0
Evening time (17:00-23:59)	40.2	43.1	47.8

1 The sample sizes (n) represent the number of observations in the data, while the proportions (%) account for the sampling weights.

**Table 2.** Characteristics of participants according to three clusters of temporal dietary patterns^1^

Characteristics	Total (n=8,147)	Cluster 1 (n=3,334)	Cluster 2 (n=2,581)	Cluster 3 (n=2,232)	p-value^2^
Gender					0.035
Men	3,588 (53.5)	1,410 (51.6)	1,155 (54.0)	1,023 (55.6)	
Women	4,559 (46.5)	1,924 (48.4)	1,426 (46.0)	1,209 (44.4)	
Age (yr)					<0.001
20-34	1,892 (30.7)	583 (24.0)	670 (34.0)	639 (35.9)	
35-49	3,044 (36.5)	1,151 (35.1)	998 (37.0)	895 (37.9)	
50-65	3,211 (32.8)	1,600 (40.9)	913 (29.0)	698 (26.2)	
Education					<0.001
Middle school or less	1,378 (13.4)	687 (16.5)	369 (11.3)	322 (11.6)	
High school	2,830 (38.2)	1,114 (37.0)	928 (39.5)	788 (38.4)	
College graduate or higher	3,596 (48.4)	1,366 (46.5)	1,191 (49.2)	1,039 (50.0)	
Household income					0.517
Lowest	788 (9.3)	339 (9.8)	246 (9.2)	203 (8.8)	
Lower middle	1,973 (23.1)	801 (23.1)	621 (23.3)	551 (22.8)	
Upper middle	2,550 (31.7)	1,038 (31.3)	836 (32.9)	676 (30.7)	
Highest	2,825 (35.9)	1,151 (35.6)	875 (34.6)	799 (37.7)	
Current smokers	1,700 (24.6)	590 (21.0)	565 (25.4)	590 (28.7)	<0.001
Current drinkers	4,903 (64.4)	1,859 (60.2)	1,578 (64.5)	1,859 (69.9)	<0.001
Participating in aerobic exercise	3,485 (47.8)	1,416 (48.1)	1,112 (47.7)	1,416 (47.5)	0.919
Feeling stressed	2,286 (29.1)	861 (27.1)	741 (29.6)	861 (31.3)	0.014

Values are presented as number (%).

1 The sample sizes (n) represent the number of observations in the data, while the proportions (%) account for the sampling weights.

2 From a chi-square test that considers the sampling weights.

**Table 3.** Nutrition intake according to three clusters of temporal dietary patterns^1^

Nutrition intakes	Total	Cluster 1	Cluster 2	Cluster 3	p-value
Total energy intake (kcal)	2,118.5±12.0	2,150.3±15.3^a^	2,020.1±20.0^b^	2,188.2±22.2^a^	<0.001
Protein (%E)	14.5±0.1	14.3±0.1	14.6±0.1	14.6±0.1	0.14
Carbohydrates (%E)	60.0±0.2	63.0±0.2^a^	60.7±0.3^b^	55.0±0.4^c^	<0.001
Total sugar (%E)	12.5±0.1	13.1±0.2^a^	12.6±0.2^a^	11.7±0.2^b^	<0.001
Fat (%E)	20.5±0.1	19.2±0.2^a^	20.4±0.2^b^	22.2±0.3^c^	<0.001
Saturated fat (%E)	6.7±0.0	6.3±0.1^a^	6.7±0.1^b^	7.2±0.1^c^	<0.001
Sodium (mg)	3,671.2±29.1	3,726.1±39.9^a^	3,575.7±49.6^b^	3,706.4±50.9^a,b^	0.033
Sodium density/1,000 kcal (mg)	1,763.6±11.0	1,750.5±15.1^a^	1,807.0±20.3^b^	1,731.4±17.9^a^	0.012
Fiber (g)	25.0±0.2	27.1±0.3^a^	24.3±0.3^b^	23.1±0.4^c^	<0.001
Fiber density/1,000 kcal (g)	12.3±0.1	13.0±0.1^a^	12.5±0.1^b^	11.1±0.1^c^	<0.001
Total Healthy Eating Index	61.5±0.2	64.9±0.3^a^	60.5±0.3^b^	58.2±0.3^c^	<0.001
Adequacy items					
Breakfast	6.5±0.1	7.4±0.1^a^	6.0±0.1^b^	5.9±0.1^b^	<0.001
Whole grain	1.7±0.0	2.0±0.1^a^	1.6±0.1^b^	1.4±0.1^c^	<0.001
Fruits (any type)	1.9±0.0	2.2±0.1^a^	1.9±0.1^b^	1.7±0.1^c^	<0.001
Fruits (fresh only)	2.2±0.0	2.4±0.1^a^	2.1±0.1^b^	1.9±0.1^c^	<0.001
Vegetables (any type)	3.5±0.0	3.6±0.0^a^	3.4±0.0^b^	3.3±0.0^c^	<0.001
Vegetables (excluding pickled)	3.1±0.0	3.2±0.0^a^	3.0±0.0^b^	3.1±0.0^a,b^	0.001
Protein sources	7.3±0.0	7.4±0.1^a^	7.2±0.1^b^	7.4±0.1^a^	0.019
Dairy products	3.4±0.1	3.8±0.1^a^	3.1±0.1^b^	3.1±0.1^b^	<0.001
Subtotal	29.7±0.2	32.0±0.2^a^	28.4±0.3^b^	27.9±0.3^b^	<0.001
Moderation items					
Saturated fat (%E)	7.0±0.1	7.5±0.1^a^	7.1±0.1^b^	6.3±0.1^c^	<0.001
Sodium	6.4±0.1	6.3±0.1^a^	6.6±0.1^c^	6.4±0.1^b^	0.031
Sugar (%E)	9.0±0.0	9.0±0.1	8.9±0.1	9.1±0.1	0.089
Subtotal	22.4±0.1	22.8±0.1^a^	22.5±0.1^a^	21.7±0.1^b^	<0.001
Balance of energy items					
Carbohydrates (%E)	2.8±0.0	2.9±0.0^a^	2.9±0.1^a^	2.5±0.1^b^	<0.001
Fat (%E)	3.6±0.0	3.7±0.0^a^	3.7±0.0^a^	3.3±0.1^b^	<0.001
Energy adequacy	3.1±0.0	3.5±0.0^a^	3.0±0.1^b^	2.8±0.1^c^	<0.001
Subtotal	9.5±0.1	10.0±0.1^a^	9.6±0.1^b^	8.6±0.1^c^	<0.001

Values are presented as mean±standard deviation. %E, percentage of energy.

1 Means and p-values are derived from generalized linear models accounting for age and gender post-hoc analyses with Bonferroni adjustment are conducted to measure significances in mean differences among clusters; Different superscription(a, b, c) denotes statistically differences between groups at 0.05 level.

**Table 4.** The metabolic indicators according to three clusters based on the timing of energy intake^1^

Metabolic indicators	Total	Cluster 1	Cluster 2	Cluster 3	p-value
BMI (kg/m^2^)	23.6±0.1	23.4±0.1^a^	23.7±0.1^b^	23.8±0.1^b^	0.001
WC (cm)	81.2±0.1	80.6±0.2^a^	81.3±0.2^b^	81.7±0.2^b^	0.001
SBP (mmHg)	115.4±0.2	114.6±0.3^a^	115.9±0.3^b^	116.0±0.4^b^	0.001
DBP (mmHg)	76.7±0.1	75.8±0.2^a^	77.3±0.2^b^	77.2±0.2^b^	<0.001
FBG (mg/dL)	97.5±0.2	97.1±0.4	97.8±0.4	97.7±0.5	0.490
TGs (mg/dL)	139.6±1.7	132.6±2.2^a^	140.3±2.6^a,b^	148.3±3.8^b^	0.001
T-chol (mg/dL)	194.9±0.5	193.3±0.7^a^	195.3±0.8^a,b^	196.5±0.8^b^	0.005
LDL-chol (mg/dL)	115.3±0.4	114.7±0.6	115.8±0.8	115.3±0.9	0.542
HDL-chol (mg/dL)	51.7±0.2	52.0±0.2	51.4±0.3	51.5±0.3	0.207

Values are presented as mean±standard deviation. BMI, body mass index; WC, waist circumference; SBP, systolic blood pressure; DBP, diastolic blood pressure; FBG, fasting blood glucose; TGs, triglycerides; T-chol, total cholesterol; LDL-chol, low-density lipoprotein cholesterol; HDL-chol, high-density lipoprotein cholesterol.

1 Means and p-values are derived from generalized linear models accounting for age, gender, education level, current smoking status, current drinking status, and participating in aerobic exercise; Post-hoc analyses with Bonferroni adjustment are conducted to measure significances in mean differences among clusters; Different superscription(a, b, c) denotes statistically differences between groups at 0.05 level.

**Table 5.** Odd ratios^1^ of obesity and MetS of three clusters of temporal dietary patterns

Variables	Cluster 1	Cluster 2	Cluster 3
Obesity (BMI ≥25 kg/m^2^)	1.00 (reference)	1.12 (0.97, 1.30)	1.19 (1.03, 1.37)
MetS	1.00 (reference)	1.26 (1.08, 1.48)	1.37 (1.17, 1.61)
Components of MetS			
Central obesity: WC ≥90 cm (men), 85 cm (women)	1.00 (reference)	1.10 (0.94, 1.29)	1.12 (0.96, 1.31)
Elevated TGs	1.00 (reference)	1.08 (0.93, 1.25)	1.25 (1.08, 1.45)
Low HDL-chol	1.00 (reference)	1.16 (1.00, 1.34)	1.13 (0.98, 1.30)
Elevated blood glucose	1.00 (reference)	1.09 (0.94, 1.27)	1.25 (1.07, 1.47)
Elevated blood pressure	1.00 (reference)	1.18 (1.02, 1.37)	1.22 (1.04, 1.43)

Values are presented as odds ratio (95% confidence interval). MetS, metabolic syndrome; BMI, body mass index; WC, waist circumference; TGs, triglycerides; HDL-chol, high-density lipoprotein cholesterol.

1 Odds ratios are obtained from logistic regression models adjusting for age, gender, education level, current smoking status, current drinking status, participating in aerobic exercise, total energy intake and Healthy Eating Index.
