# Supplementary material for: Higher energy consumption in the evening is associated with increased odds of obesity and metabolic syndrome: findings from the 2016-2018 Korea National Health and Nutrition Examination Survey (7th KNHANES)
Source: Epidemiol Health. 2023 Sep 19;45:e2023087. doi: 10.4178/epih.e2023087 (PMC10867517; doi:10.4178/epih.e2023087)
Supplement: Supplementary Material 1. [file epih-45-e2023087-Supplementary-1.docx]

**Supplementary Material 1**

**Methods**

**Metabolic Syndrome (MetS) diagnosis**

In the current study, MetS was diagnosed according to the adult criteria of the International Diabetes Federation (IDF) (1), which defines MetS as meeting at least three out of five criteria. The MetS diagnostic criteria provided by the IDF are as follows:

1. Waist circumference ≥90 cm for men or ≥85 cm for women
2. Systolic BP of 130 mmHg of Diastolic BP of 85 mmHg or treatment with anti-hypertensive medication
3. TGs ≥150 mg/dL or taking dyslipidemia drugs
4. HDL-chol <40 mg/dL for men or <50 mg/dL for women
5. Fasting plasma glucose (FPG) ≥100 mg/dL or or treatment of previously diagnosed type 2 diabetes mellitus
