# Supplementary material for: Higher energy consumption in the evening is associated with increased odds of obesity and metabolic syndrome: findings from the 2016-2018 Korea National Health and Nutrition Examination Survey (7th KNHANES)
Source: Epidemiol Health. 2023 Sep 19;45:e2023087. doi: 10.4178/epih.e2023087 (PMC10867517; doi:10.4178/epih.e2023087)
Supplement: Supplementary Material 2. [file epih-45-e2023087-Supplementary-2.docx]

**Supplementary Material 2**

**Table S1: Values of cluster validity indices**

|  | 2 | 3 | 4 | 5 | 6 | 7 | 8 | 9 | 10 |
| --- | --- | --- | --- | --- | --- | --- | --- | --- | --- |
| Silhouette index | 0.1923 | 0.1628 | 0.1619 | 0.1679 | 0.1787 | 0.1157 | 0.1521 | 0.1419 | 0.1538 |
| Dunn index | 0.0043 | 0.0053 | 0.0044 | 0.0044 | 0.0039 | 0.0014 | 0.0043 | 0.0009 | 0.0034 |
| Calinski-Harabasz index | 5432.  5462 | 3449.  2115 | 2512.  9421 | 1884.  4187 | 1558.  0030 | 1338.  7598 | 1882.  2194 | 1158.  7263 | 941.  0703 |
| Score Function | 0.1367 | 0.0599 | 0.0321 | 0.0152 | 0.0053 | 0.0042 | 0.0013 | 0.0008 | 0.0002 |
| Davies-Bouldin index | 1.4090 | 1.5650 | 1.8396 | 1.9111 | 1.6885 | 2.2963 | 2.2166 | 1.8596 | 1.9021 |
| Modified Davies-Bouldin index | 1.4090 | 1.6295 | 2.0654 | 2.1354 | 1.9080 | 2.7509 | 2.5823 | 2.2298 | 2.3043 |

**Figure S1: Optimal number of clusters determined by indices' maximum value**


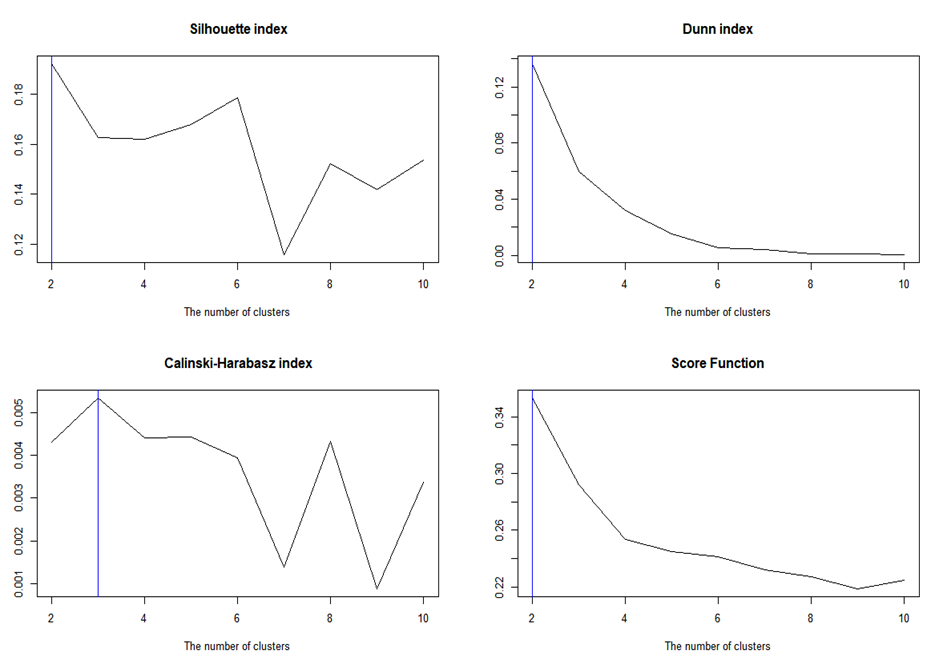


**Figure S2: Optimal number of clusters determined by indices' minimum value**

**
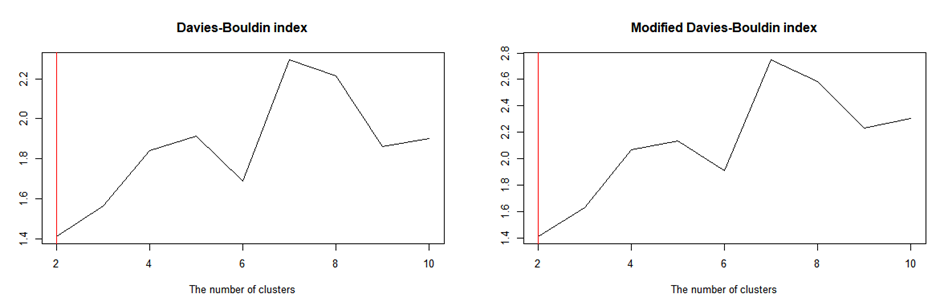
**
