# Supplementary material for: Higher energy consumption in the evening is associated with increased odds of obesity and metabolic syndrome: findings from the 2016-2018 Korea National Health and Nutrition Examination Survey (7th KNHANES)
Source: Epidemiol Health. 2023 Sep 19;45:e2023087. doi: 10.4178/epih.e2023087 (PMC10867517; doi:10.4178/epih.e2023087)
Supplement: Supplementary Material 3. [file epih-45-e2023087-Supplementary-3.docx]

**Supplementary Material 3**

**Methods**

**Temporal dietary patterns (TDPs)**

The cluster initial value of the Kernel k-means clustering was set from 2 to 10, and the optimal number of clusters was determined using cluster validity indices as the performance measure. Performance of cluster analysis is evaluated based on two criteria: cohesion and separation. Cohesion refers to the density of data points within each cluster, while separation measures the distance between clusters. The fundamental principle of clustering is to maximize the cohesion within the same cluster while also maximizing the separation from other clusters. These two criteria are combined to create a single metric known as cluster validity index. The pursuit of effective cluster validity indices has been a topic of significant research. In this study, 6 representative indices were used as cluster validity indices: Silhouette index, Dunn index, Calinski-Harabasz index, and Score Function identify the optimal number of clusters at the point where the metric reaches its maximum value. Conversely, Davies-Bouldin index and Modified Davies-Bouldin index determine the optimal number of clusters at the point where the metric reaches its minimum value. Figure S1 and Figure S2 in the Supplementary Material 2 represent the values of each indices graphically, according to the number of clusters, and indicate the maximum or minimum values. Table S1 in the Supplementary Material 2 shows the values of cluster validity indices. According to the metrics, we can conclude that 2 to 3 clusters are appropriate. Cluster analysis was conducted on the distribution of the proportion of caloric intake over a 24-hour, divided into the time points of morning, lunch, and dinner. As a result, three distinct clusters were obtained.

**References**

1. Federation ID. The IDF consensus worldwide definition of the metabolic syndrome. International Diabetes Federation: International Diabetes Federation; 2006 [cited 2023 May 3]. Available from: <https://idf.org/media/uploads/2023/05/attachments-30.pdf>.
